# Research Priorities for NCD Prevention and Climate Change: An International Delphi Survey

**DOI:** 10.3390/ijerph121012941

**Published:** 2015-10-16

**Authors:** Ruth Colagiuri, Sinead Boylan, Emily Morrice

**Affiliations:** 1 Menzies Centre for Health Policy, School of Public Health, The University of Sydney, Sydney, NSW 2006, Australia; E-Mail: ruth.colagiuri@sydney.edu.au; 2 The Boden Institute of Obesity, Nutrition, Exercise & Eating Disorders, Charles Perkins Centre, The University of Sydney, Sydney, NSW 2006, Australia; E-Mail: sinead.boylan@sydney.edu.au

**Keywords:** non-communicable disease, climate change, research priorities, agenda setting

## Abstract

Climate change and non-communicable diseases (NCDs) are arguably the greatest global challenges of the 21st Century. However, the confluence between them remains under-examined and there is little evidence of a comprehensive, systematic approach to identifying research priorities to mitigate their joint impact. Consequently, we: (i) convened a workshop of academics (n = 25) from the Worldwide Universities Network to identify priority areas at the interface between NCDs and climate change; (ii) conducted a Delphi survey of international opinion leaders in public health and relevant other disciplines; and (iii) convened an expert panel to review and advise on final priorities. Three research areas (water security; transport; conceptualising NCD harms to support policy formation) were listed among the top 10 priorities by >90% of Delphi respondents, and ranked among the top 12 priorities by >60% of respondents who ranked the order of priority. A fourth area (reducing the carbon footprint of cities) was ranked highest by the same >60% of respondents. Our results are consistent with existing frameworks on health and climate change, and extends them by focusing specifically on NCDs. Researching these priorities could progress understanding of climate change and NCDs, and inform global and national policy decisions for mitigating associated harms.

## 1. Introduction

The world is experiencing a triple crisis of chronic, non-communicable diseases (NCDs), economic and geopolitical instability and climatic volatility. The magnitude of these crises is unprecedented with both climate change [1] and NCDs [2] described as among the 21st Century’s most daunting challenges.

The world awoke slowly to the threat of climate change, defined as “…a change in the state of the climate that can be identified by changes in the mean and/or the variability of its properties, and that persists for an extended period, typically decades or longer” [3]. There is now a compelling body of literature on this phenomenon which we will not report on here. Suffice to say that the strength of the evidence is such that the UN; governments; corporate, church, academic and global health leaders now accept the reality of climate change, and are increasingly aware of its potentially dire consequences for human health and development.

Similarly, the threat of NCDs was not well-perceived until the major NCDs—cardiovascular disease; diabetes; cancer; and chronic respiratory diseases—already accounted for >60% of the world’s annual deaths [4] and by 2030 the toll will rise to 75% of global deaths [5]. The morbidity, mortality, health system and societal implications of this are well documented elsewhere, for example there have been four Lancet Series on NCDs since 2005 [6,7,8,9]. The macro-economic consequences of NCD-related illness and disability from absenteeism, early retirement, and premature death foster national and global economic uncertainty and limit economic growth [4,5,10,11] and, thus, hinders poverty alleviation and human development. This burden disproportionately affects developing countries which have the least capacity to respond. Likewise with climate change, 99% of the projected associated health burden will be borne by the developing world despite it generating only 3% of carbon emissions [12].

The magnitude of the global impact of NCDs is such that, in 2011, The UN General Assembly held its second only special meeting dedicated to a specific disease area on NCDs—the first being on HIV/AIDS 10 years earlier. The UN High Level Meeting on NCDs resulted in a Political Declaration [13] which called for action on NCDs and noted issues such as unhealthy urbanization and unhealthy diets as contributing factors to their rapid escalation. Shortly thereafter, the UN Rio+20 Conference on Sustainable Development published its Outcomes Document, *The Future We Want* [2] which recognizes health as: “*a precondition for and an outcome and indicator of all three dimensions of sustainable development….*” the three dimensions being environmental, social and economic. The Rio+20 Outcomes Document also specifically cites NCDs as a threat to sustainable development.

Given this it is surprising that the links between climate change and NCDs has received relatively little attention to date. However, there is a sizeable and growing literature on climate change and health more broadly. For example, the notion of ‘one planet-one health’ holds that human, animal, and planetary health are so inextricably interlinked that the well-being of each is dependent on the well-being of the other [14,15]. Two Lancet series [16,17] outline a litany of climate change impacts on health from heat stress, floods, droughts and storms, as well as less direct impacts such as changing infectious disease patterns, food insecurity, and air pollution. Interestingly there is also emerging evidence of links between NCDs and some of the major infectious diseases [18]. While the Lancet series includes some discussion of NCDs, the WHO’s quantitative risk assessment of the effect of climate change on selected mortality [19] focusses on heat- and flood- related mortality, diarrheal diseases, under-nutrition, malaria and Dengue.

NCDs are not as obviously associated with climate change as are fires, floods, famines, changes in the distribution of existing infectious diseases and the emergence of new microbial infections. But, on closer examination, increases in obesity and other risks for NCDs over recent decades have much in common with the causes of environmental degradation. Genes and ageing aside, the major NCDs share four common, modifiable risks, *i.e.*, inappropriate diets (under- or over-nutrition), physical inactivity, tobacco use, and alcohol misuse. While the last two of these are largely socially mediated, the first two are closely linked to causes of climate change. This is not to say that climate change and NCDs cause each other, although in some instances there may be causal interactions. Rather, we, and others, argue that NCDs and climate change share common vectors and strong associations.

For example, the way and what we eat, how we work predominantly in cities at sedentary tasks, and transport ourselves with little physical effort, not only damages our health but also harms the planet. Some common vectors of NCDs and climate change are seen in links between greenhouse gas emissions and body mass index [20]; car travel, obesity and air pollution [21], sitting for long periods and increased risk of mortality [22]. There are also reports that soil degradation resulting from mass food production and poor land management is depleting the micro-nutrient content of fruit and vegetables [23,24,25] thus further undermining human and animal nutrition and, therefore, health. A more direct link between climate change and NCDs is the increased incidence of cardiovascular and respiratory illnesses caused by air pollution—air pollution being both *caused by* and a *cause of* climate change [17]. For example, air pollution from bush fires could be said to be caused by climate change, but air pollution from vehicle emissions is a contributing factor to climate change as well as a cause of chronic respiratory disease [26].

Despite a substantial body of frameworks to guide research, action and policy on *health* and climate change [17,27,28,29,30,31,32,33,34] there is little evidence about the effectiveness of interventions aimed at redressing their adverse interactions. Notwithstanding research priorities [35] and priority actions [36] for NCDs alone, there is even less evidence specifically about the interaction between *NCDs* and climate change.

The available literature on health and climate change is largely confined to “issues-raising” expert opinion and commentary; estimates and modelling; and reviews of non-empirical work and the “grey literature”. Such contributions are clearly important [31] but have not yet translated into comprehensive systematic, evidence-generating enquiry and there has been little emphasis on combining legal and public health thinking and solutions at the interface between NCDs and climate change [37]. Nor have the “health in all policies” [38] and health impact assessment [39] movements yet successfully bridged this divide or integrated with environment impact theory and practice, although this may be imminent [40].

As pointed out by Haines and McMichael [41], the clear definition of precisely what needs to be researched is hindered by uncertainties about the exact nature and rate of climate change and the sheer magnitude and complexity of the systems, sectors and politics involved. McMichael also detailed the challenge elsewhere [31,42], especially the need for new research methods and models and the imperative to stop applying old thinking and tools to new and unchartered problems. To deal effectively with complex issues, Finegood *et al.* [43] advised that “*…we need to move from an analysis of the determinants or causes of the problem to a solution orientation; the frameworks used to describe the problem may not be the right ones for building the "best" solutions*”.

To contribute to knowledge and understanding of the problem and attempt to move closer to a solution orientation, we used qualitative research to take a further step towards identifying priority research and action areas at the interface between NCDs and climate change, particularly those areas that might be amenable to mitigation via legal interventions. We began by anchoring the process to an existing advocacy framework which had previously been endorsed by a consensus of 100 international experts who attended the Oxford Health Alliance’s 2008 Summit in Sydney, Australia (see the Sydney Resolution at Figure S1) which called for healthy food, physical activity, work, business and policy environments. By systematically obtaining expert opinion from a range of disciplines, sectors and settings we identified a set of priority areas and questions which have potential to assist solutions-oriented, evidence-generating research to inform policy, planning and funding decisions.

### Objective

To systematically identify a priority research areas/questions at the interface between climate change and the prevention of the major NCDs.

## 2. Experimental Section

**Figure 1:**
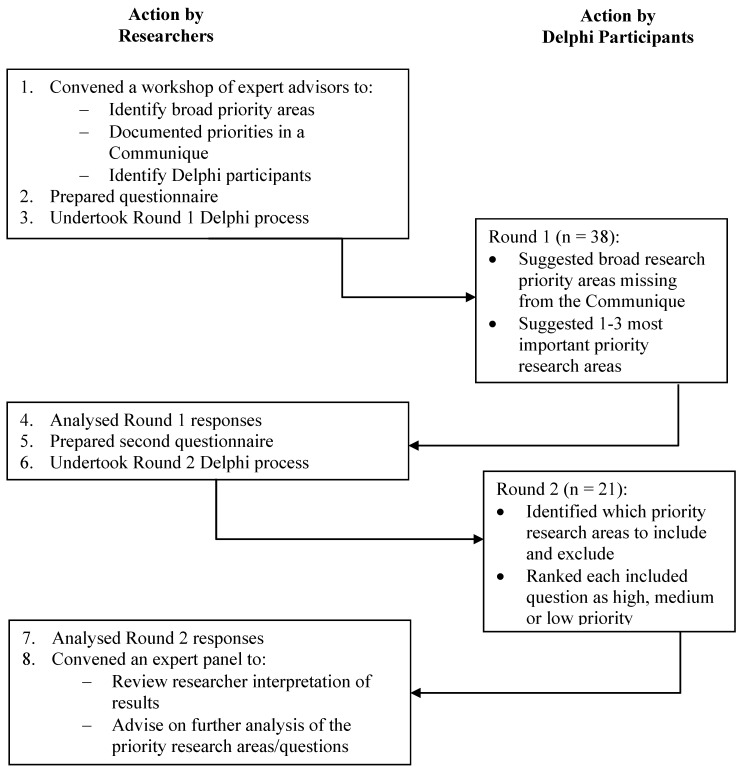
A summary of the study method and major actions.

Following approval from the University of Sydney Human Ethics Research Committee (reference number 13885) a three step process (Figure 1) was undertaken.

### 2.1. Step 1: Initial Identification of Priority Research Areas

We convened a three day workshop at The University of Sydney under the auspices of the Global Health Justice Network of the Worldwide Universities Network (WUN). The aim was to explore, debate and identify broad areas of knowledge deficit and priorities at the interface between NCDs and climate change to which the law might be applied to mitigate current and/or future harms and achieve co-benefits. The workshop followed a structured process of presentations by WUN members on various aspects of NCDs and climate change, panels, facilitated plenary debate, and small group discussions. The 2008 Sydney Resolution [44], a call to action on NCDs acknowledging the link with climate change and advocating for healthy places, healthy food, healthy business, healthy public policy and healthy societies, was used as the framework and starting point for the discussions. The plenary debate and small group discussion sessions were documented during the workshop.

Broad areas for research were identified as priorities if they:

(a) had the capacity to impact positively one or more negative aspects of the interface between climate change and NCDs
(b) were amenable to mitigation through legal and ethical frameworks and governance systems
(c) had the potential to address critical deficits in our knowledge and understanding of how to mitigate and/or manage effects of the current NCD and climate change crises.

The results were documented in a workshop consensus Communiqué (Figure S2)

### 2.2. Step 2: An International Delphi Process

#### 2.2.1. Identification of Priority Research Questions

A Delphi process [45] was used to test and expand on the priority areas identified through the workshop.

#### 2.2.2. Delphi Participants

Participants were suggested by the workshop delegates, from among authors of relevant journal articles, and professional contacts of one of the authors (RC) from among experts involved in the development of the Sydney Resolution [44]. We sought participants from a wide geographical spread, both genders, a range of ages and career stages, and a variety of disciplines and sectors.

#### 2.2.3. Data Collection

The Delphi process consisted of two rounds and was completed in 2012. In **Round 1**: the Communiqué comprising the workshop delegates’ priorities, a participant information sheet and a semi-structured questionnaire were emailed to an international sample of experts who had not attended the workshop. The questionnaire asked participants to: (i) state what, if any, broad research priority areas they believed were missing from the Communiqué; and (ii) suggest at least one but no more than three priority research questions that would address one or more knowledge deficits in the overlap between climate change and NCD prevention and which would be feasible to conduct (see questionnaire at Figure S3).

The **Round 2** questionnaire consisted of the responses to Round 1 after removal of duplicates and arranged in categories. It was emailed with an explanatory letter and instructions to respondents from Round 1 who were asked to either: (i) exclude or include the priority research areas identified in Round 1; and (ii) to rank each included question as either high, medium or low priority (see questionnaire at Figure S4). Specifically, participants were requested to identify those areas and questions that:

- were of central importance to the global response to NCD prevention and climate change;
- addressed a knowledge or evidence deficit that is preventing progress in an area of high importance to the overlap between mitigating or adapting to climate change and preventing or significantly reducing the impact of NCDs and/or which focused on social and health justice issues;
- addressed problems at the interface between NCDs and climate change which may be amenable to modification through legal interventions;
- had a degree of urgency or immediacy.

#### 2.2.4. Analysis

Two of the authors (SB and RC) reviewed the responses to Round 1, removed duplicates and collated the remaining responses under 13 categories: food production; food security; policy; corporate social responsibility; urban design, transport and housing; regulatory interventions; social justice; economics; trade; behaviour and communication; information; systems; and water. The responses were then assessed for quality and consistency by three academic experts in health policy and law, sociology and politics from Bristol University (UK) who had attended the workshop. The categories were further condensed and each of the 100 questions gleaned from Round 1 was allocated to one only of six categories: food systems and security; urban design, transport and housing; economics trade and business; social justice; behaviour, communication and information systems; and regulation, governance and policy, which formed the structure of the Round 2 questionnaire.

Responses from Round 2 were entered onto an Excel spreadsheet and analyzed by the:

- majority agreement to include as a *high* priority;
- ranking of order of importance as a *high* priority;
- majority agreement to identify as a *low* priority.

### 2.3. Step 3: Verification and Deconstruction of the Final Priority Questions

Following analysis of the responses to Round 2, an expert panel of three internationally recognized academics reviewed the researchers’ interpretation and rankings of the final results and advised on further analysis of the priority areas/questions.

## 3. Results

### 3.1. Workshop Participants

The workshop was attended by 25 WUN academics from Australia, Canada, the United States, the United Kingdom, and the West Indies, and three NCD non-government organisations. The disciplines represented included environmental health, epidemiology, ethics, climate change, health policy, nutrition, public health, law, psychology, philosophy, sociology, and politics.

### 3.2. Workshop Output

The key output of the workshop was a Communiqué (Figure S2) outlining five broad priority research areas identified by delegates at the interface between NCDs and climate change. They were: (i) global and national policy; (ii) food systems; (iii) corporate social responsibility; (iv) health in all policies; and (v) specific regulatory interventions.

### 3.3. Delphi Process

As illustrated in Table 1, Delphi participants were from a range of geographical locations and disciplines. The non-government, not-for-profit organisations were the International Diabetes Federation, International Union for Cancer Control and the Australian Heart Foundation.

### 3.4. Round 1 Results

One hundred and six participants were included in Round 1 and 46 (43%) responded. Eight respondents excluded themselves on the basis of ill-health, limited access/time or inadequate expertise. This left 38 (36%) respondents who submitted a completed response.

After removal of duplicates from Round 1, 100 potential priority research areas and questions (Figure S3) remained that fell into six broad categories—*i.e.*, food systems and security; urban design, transport and housing; economics trade and business; social justice; behaviour, communication and information systems; and regulation, governance and policy.

### 3.5. Round 2 Results

The questionnaire for Round 2 was emailed to the 38 respondents from Round 1 and yielded a 55% (n = 21) response rate. Ten research areas/questions on aspects of water security, food and agriculture, transport, urbanisation, planning and design, taxation, incentives and disincentives were identified by > 90% of respondents as a research priority (Table 2).

**Table 1:** Delphi process participant characteristics.

Characteristic	Round 1	Round 2
	n-size	n-size
Response rate	46	22
**Subjects**		
Male / Female	30/8	15/6
**Location**		
Europe	18	9
Oceania	8	5
United States	5	4
Caribbean	2	1
Canada	1	-
Africa	1	1
South America	1	1
India	1	-
South East Asia	1	-
**Discipline**		
Public health(incl.epidemiology)	21	11
Law (public health/social justice)	4	3
Geography	3	2
Agriculture	2	2
Transport	2	1
Policy	1	1
Nutrition	1	1
Environmental health	1	-
Planning (urban)	2	-
Economics	1	-
**Sector**		
Academia	22	10
Not-for profit organisation	3	3
Government	12	7
Industry	1	1

**Table 2:** Agreement (>90%) to include question as a research priority. NB: Questions also presented in Table 3 are in bold.

Question	Area	n	%
**Access to potable water for human consumption and agriculture: what current best practices are needed to provide equitable access to water between geographic regions and usage demands? Are new best practices needed given climate change?**	F	20	95
How do national agricultural policies affect urban food security in different regions?	F	20	95
What are the most effective ways to shift consumer demand from the “western” diet to a more diverse predominantly plant-based diet?	BCI	20	95
What are the likely environmental effects of a fat tax/healthy subsidy to encourage healthier diet?	RGP	20	95
**What are the most appropriate policies to shift from private motorized transport to healthier and cleaner transport alternatives?**	U	19	90.5
**How can the harms that lead to NCDs be described and conceptualised in a way that more easily supports policy formation?**	BCI	19	90.5
How can housing policies contribute to climate change adaptation and mitigation?	U	19	90.5
What are the methodologies to measure the impact of sustainable urban development and transport on health?	U	19	90.5
What are effective incentives and disincentives for healthy and unhealthy food consumption which also promote low-carbon solutions?	F	19	90.5
What metrics, policies and governance structures are needed to address tough trade-offs needed in shifting agricultural patterns underway, especially aimed at reducing long term demand for meat and palm and developing a more diverse diet?	F	19	90.5

Key: BCI: Behaviour, communication and information systems; F: Food; RGP: Regulation, governance, policy; U: Urban design, transport and housing.

Not all respondents ranked the order of importance of all the questions they indicated as priorities. However, 12 research areas/questions were rated highest by the 60% of Round 2 respondents who ranked their included priorities (Table 3). Three of these coincided with the priorities identified by over 90% of the participants (Table 2) and concerned:

- best practices for access to potable water
- policies for shifting motorised transport to cleaner healthy transport
- describing NCDS in a way that supports policy change

The highest ranked question (Table 3) was: “*How can car-dependent cities shrink their carbon footprints in a way that reduces health inequalities?*” thus providing a fourth high priority from the combined 19 priority areas illustrated in Table 2 and Table 3.

**Table 3:** Agreement (>60%) to rank question as high priority. NB: Questions also presented in Table 2 are in bold.

Question	Area	n	%
How can car-dependent cities shrink their carbon footprints in a way that reduces health inequalities?	U	12	75
What are the cost-benefits of fiscal policies such as a fat tax/ healthy subsidies, taking into account the potential benefits or costs that they might have in terms of environmental effects?	ETB	9	75
**Access to potable water for human consumption and agriculture: what current best practices are needed to provide equitable access to water between geographic regions and usage demands? Are new best practices needed given climate change?**	F	13	68
Which strategies are the most appropriate to improve food security through small-scale farming and environmentally sustainable food production?	F	11	65
How can the health and climate change agendas be better aligned with those of urban planners and real estate developers?	U	11	65
**What are the most appropriate policies to shift from private motorized transport to healthier and cleaner transport alternatives?**	U	11	65
How can food needs be defined to meet optimal nutrition within an environmentally sustainable agricultural program?	F	9	64
How can we effectively predict crop production changes due to climate change and effects on food security (crop adaptation in regions most likely affected)?	F	9	64
What joint mechanisms can countries put in place to reduce the negative impacts of trade on climate change and NCDs?	ETB	9	64
How to change behaviour (as a collective rather than as individuals) to a highly active, low carbon transport system- (a question for primary research and for systems modelling). How to minimise rebound effects for a behaviour change?	BCI	10	62.5
How to develop evaluation tools for social decision making that go beyond cost benefit analysis approaches?	RGP	10	62.5
**How can the harms that lead to NCDs be described and conceptualised in a way that more easily supports policy formation?**	BCI	11	61

Key: BCI: Behaviour, communication and information systems; ETB: Economics, trade and business; F: Food; RGP: Regulation, governance, policy; U: Urban design, transport and housing.

Nine questions were agreed by >90% of respondents to be excluded as a priority research question (Figure S5). A further five questions were ranked as “low priority” with >60% agreement amongst respondents (Figure S6). Four of these questions were concerned with information and communication issues. Three of them were also were also agreed to be *excluded* by >90% of respondents. Nonetheless, it is not clear if they were ranked low/excluded because of their content focus or their lack of potential to be effectively researched.

## 4. Discussion

This study identified a set of priority areas and questions which can assist in guiding research and action aimed at mitigating the joint impacts of NCDs and climate change. Of the total 19 priorities identified, the three high priority questions common to both Table 2 (>90% agreed to include) and Table 3 (>60% agreed as a “high priority”) plus the highest ranked priority (Table 3), can be used reasonably confidently as a basis for future research. The process of identifying high priorities (Table 2 and Table 3) had the advantage of concomitantly highlighting low priorities (Figures S5 and S6).

The identified priorities are consistent with existing frameworks and position statements on climate change and health but are considerably more detailed. In particular they reflect many aspects of the WHO research priorities consensus [27] which presents five empirical and scenario-based categories *i.e.*, (i) assessing the risk; (ii) identifying effective interventions; (iii) guiding health-promoting mitigation and adaption decisions in other sectors; (iv) improving decision support; and (v) estimating the costs of protecting health from climate change. Moreover, the priority question, “*how can the harms that lead to NCDs be described and conceptualised in a way that more easily supports policy formation?*”, fits well with the proposition that, there is a premium on “*understanding (among other issues) how best to frame advice for policy-makers…*” [31].

The need for research on climate change and food security and systems, and urban design, transport and housing, dominated the identified priorities. Behaviour, communication and information systems were also prominent. Further, all the priority areas and questions met the criteria of: having the capacity to impact positively one or more negative aspects of the interface between climate change and NCDs; being amenable to mitigation through legal and ethical frameworks and governance systems; and the potential to address critical deficits in our knowledge and understanding of how to mitigate and/or manage impacts of the NCD and climate change crises.

The Delphi process was a suitable method for surveying experts on this topic. It was similarly used previously to achieve expert agreement on “grand challenges” for NCD prevention [35], many of which are consistent with the priorities identified in our study. The response rate was acceptable [45], and ranking is one of the most common measures used to identify consensus in the Delphi process [46]. The Round 1 questionnaire was informed by a summary of the results of face-to-face debate and careful consideration of the issues by a panel of 25 multi-disciplinary, international experts and stakeholders. It was structured yet flexible which is reportedly an advantage [47,48]. Moreover, there were inbuilt validations of the results. For example: three external experts reviewed the analysis of Round 1; another expert panel reviewed the final results; the 10 priorities identified in Table 2 had a high rate of respondent agreement (>90%) and three of these were confirmed as high priority by the rankings shown in Table 3.

However, inevitably there are limitations to the Delphi method such as issues with questionnaire design, the selection of experts, subject and researcher bias, and lack of representation, as well as challenges in managing larger groups [47,49]. Other methods of gaining consensus do exist including structured expert judgement [50], consensus development conference, nominal group technique [47] and brainstorming [49]. There are also different forms of Delphi methods including the “policy Delphi” and the “real time Delphi” [49]. The Delphi survey in this current study was chosen as the method of gaining consensus as its use is recommended when examining less explored topics and identifying/prioritizing issues [45,47]. In addition, exposure to subject bias may be also seen as an advantage as its presence may change other subject’s views, hence generating consensus [47,49]. Additionally, Round 2 respondents were exposed to the yield of research questions and areas from Round 1, which would likely reduce promote congruence and reduce confusion.

Participants in both the face-to-face workshop and the subsequent Delphi process came from a diverse range of disciplines and geographical regions. A number of steps were taken to reduce possible confusion and misunderstanding of the task. These included the use of the Sydney Resolution (Figure S1) as the initial framing for workshop participants, and the Communiqué from the workshop (Figure S2) being provided to Delphi respondents along with explicit instructions for the inclusion and ranking of priorities. The selection of respondents was from among experts with recognized knowledge, experience and interest in the general topic. However, the characteristics of the Delphi respondents changed between Round 1 and Round 2 in that the ratio of males to females almost halved as did the proportion of respondents from Europe versus other regions, and the proportion of academics versus other sectors (Table 1). It is not possible from our research to know what if any bias this may have introduced.

Another potential source of bias may be that representation from the developing world was limited in both the initial workshop and Delphi process, primarily because NCDs and climate change is a new field of enquiry and resources, and infrastructure and academic expertise is limited in developing countries. There was a lack of representation at the workshop from disciplines such as architecture; urban planning; transport; and economics. However, this was remedied in the selection of Delphi survey participants and it has been argued that, where few experts are available, a small sample may be used with confidence [51]. Several Round 2 respondents did not rank the research questions. Consequently, the results tend to emphasize the priority inclusion of research topics rather than their specific order of priority. Some of the proposed priorities included more than one research question and are, therefore, not readily researchable without further breakdown. The survey was completed in 2012 and it is possible that respondents’ opinions may have changed since then. Notwithstanding this, the identified priorities are not inconsistent with the 2015 recommendations of the newly formed Lancet Commission on Health and Climate Change [17]. Nor did our literature review indicate that the identified questions and areas have been satisfactorily addressed in the interim.

The extensive variety of research modalities and disciplines required to generate credible evidence to support policy solutions in this area is hindered by practical impediments including the absence of a common language and purpose for communicating and debating the confluence of NCDs and climate change. As noted by McMichael [31], population health researchers and epidemiologists primarily focus on methods and problems within their ‘comfort zone’ such as exposure to risk from infections or toxins versus their effect, and controllable versus confounding variables. He further pointed out that environmental health theory and practice is based on and directed by efforts to understand exposure and effect but, despite the sophistication of new technologies for quantifying such exposures, pales into insignificance when compared with attempting to understand and research the complexities and uncertainties implicit in climate change and health. Additionally, as shown in an audit of Canadian research on health and climate change, despite recent increases, funding is inadequate and is not commensurate with the risks posed [52]. Other barriers include the siloed way in which research funds have traditionally been acquired by funding bodies and dispersed to researchers, and the lack of clarity around framing and prioritizing explicit areas and questions to guide research efforts.

Notwithstanding its limitations, the process we undertook identified solution-oriented priority areas for research into NCDs and climate change whereas previous frameworks have largely outlined principles and broad areas. Prior work on NCDs and climate change has nominated *strategies* to prevent climate change which also have NCD co-benefits [53] while the results of our study frame potentially researchable *questions* and areas to guide evidence generation. The emphasis towards interventions that employ legal solutions was important for a number of reasons. Legislation, regulation and taxation are standard items in the public health toolbox and, as exemplified by the WHO’s Framework Convention on Tobacco Control [54], have been shown to promote robust global and national health governance mechanisms capable of effecting positive individual, population and sovereign health behavior and policy change.

As pointed out by others, making research into the health impacts of climate change manageable and meaningful requires firmly locating it in the broader agenda of global health equity [55,56], supported by multi-disciplinary collaboration using a variety of research methods [57], and empirical data on past and present events and outcomes, and scenario-based health risk projections [27].

## 5. Conclusions

This study identified a set of defined research areas and questions specifically for studying aspects of the intersection and interactions between NCDs and climate change that are consistent with but more readily actionable than existing broad frameworks for health and climate change. Researching these priorities could potentially progress understanding and action at the interface between climate change and NCDs, and help inform global and national policy decisions for mitigating associated harms. Together with existing health and climate change frameworks and recommendations, further refinement of these areas and questions could serve as a basis for research to unlock much needed information and evidence to guide global and national governance and policy decisions for mitigating the impact of this universal problem.

## Supplementary Files

Supplementary File 1
