# Supplementary material for: Research Priorities for NCD Prevention and Climate Change: An International Delphi Survey
Source: Int J Environ Res Public Health. 2015 Oct 16;12(10):12941–57. doi: 10.3390/ijerph121012941 (PMC4627009; doi:10.3390/ijerph121012941)
Supplement: Supplementary File 1 [file ijerph-12-12941-s001.pdf]

The Oxford Health Alliance

Figure S1 – The Sydney Resolution

Confronting the Epidemic of Chronic Disease

www.oxha.org

The Sydney Resolution

February 2008

Healthy People in Healthy Places on a Healthy Planet

The way we live is making people sick. It is also making our planet sick. It is not sustainable. We can do better.

The world is now facing the most serious challenges to human health. The magnitude and complexity of these challenges require the broadest alliance and partnership of stakeholder groups to confront this growing and urgent problem. Four preventable chronic diseases – heart disease/stroke, diabetes, chronic lung disease and cancer – account for 50% of the world's deaths. Their underlying causes are tobacco use, physical inactivity and poor diet.

These preventable chronic diseases are at epidemic proportions. They are increasingly affecting younger people and cause physical disability, depression, and early death. There are immense costs to society in lost productivity and increased use of health services. The epidemic threatens economic stability in developed and developing countries alike. Families striving to escape the poverty trap are pushed back into disadvantage and despair. The problem is similar to that of climate change in that it affects the whole world, is the result of our way of living and, crucially, can be reversed.

Urgent action is needed. There is a clear way forward. The four major chronic diseases can largely be prevented, but there is no simple or quick solution. To achieve real change, it is necessary to bring together dedicated stakeholders from all parts of society. The development of how we live as societies, share opportunities, interact with the natural environment and how we design our cities, transport systems, food systems, work places and housing will fundamentally determine future patterns of health and disease. We need health services focussed on prevention as well as cures and we need our world free of tobacco. We must fundamentally reshape our social and physical environments so that they are aligned with eradicating this epidemic of chronic disease.

The call to action

We call on the United Nations' agencies, governments, corporations and businesses, donor agencies, professionals, consumers, non-government organisations and employee unions, civil society and individuals to collaborate in taking urgent action to halt the devastating global impact of chronic diseases. We know that change is possible for individuals and families, communities and nations and that the change will promote economic and environmental sustainability. We need:

• **Healthy places – designing towns, cities and rural areas, which are smoke-free, and where it is easy to walk, cycle and play, with unpolluted open spaces and safe local areas that foster social interaction.**

• **Healthy food – making healthy food affordable, and available to all.**

• **Healthy business – engaging business in the agendas promoting healthy people, healthy places, healthy planet and making good health good business.**

• **Healthy public policy – formulating comprehensive, innovative and 'joined-up' legislation and social and economic policies that promote health.**

• **Healthy societies – addressing equity and socio-economic disadvantage.**

Oxford Health Alliance, 27 February 2008

See: www.oxha.org, www.3four50.com *and* Grand Challenges in chronic non-communicable disease. Nature 2007 450; 494-496

Figure S2 – WUN Communiqu é

Communiqué on
Combining Climate Change and NCD Prevention
from the 2nd WUN Global Health Justice Network Workshop
held, Sydney, Australia
5-7 May 2011

Rationale
Non-communicable diseases (NCDs) are not only a health problem, they are a sustainability and human development disaster. They are inextricably linked to the global environmental and economic crisis. Their impact on human capital is inexorable.

The United Nations General Assembly's unanimous decision in 2010 to hold a UN High Level Meeting on NCDs in September 2011 signals growing government recognition and concern about the impact of the global emergency arising from diabetes, heart disease and stroke, cancers and chronic respiratory diseases and their modifiable antecedent risks. These diseases are closely associated with mental illness and result in untold personal suffering and grave social and economic hardship. Individually and collectively they are major causes of poverty and lost productivity and pose a significant barrier, not only to human development, but also to global economic stability and prosperity.

These diseases form a substantial component of a broader global sustainability predicament that threatens economic and environmental sustainability and human capital. Their modifiable causes, particularly physical inactivity and under-or over-nutrition, go hand in hand with social inequality and with vectors of climate change such as over-urbanisation, poor urban design, sedentary automated work and play, and unhealthy food grown or manufactured through carbon intense food production methods. Many aspects of these things can be mitigated or ameliorated through laws. Yet, despite the obvious direct and indirect links between NCDs and climate change, the confluence remains under-examined in both global health and climate change research. Consequently, the Global Health Justice Network of the Worldwide Universities Network (WUN) met in Sydney on 5-7 May 2011 to consider how these issues might be addressed.

Purpose
The meeting brought together academic researchers from a range of disciplines including law, medicine and public health, the social sciences, environmental science, nutrition, psychology, policy, politics, and philosophy from academic institutions in the UK, the USA, Canada, the West Indies, and Australia. Its purpose was to explore, debate and identify key research deficits and priorities at the interface between NCDs and climate change to which the law might be applied to mitigate current and/or future harms and achieve co-benefits.

Process
Drawing on the 2008 *Sydney Resolution* (www.oxha.org) which called for urgent action on NCDs and their environmental vectors, delegates considered knowledge and evidence deficits that currently hinder progress on NCDs and climate change and exacerbate social injustice. The debate was anchored in the elements and components of optimally healthy and environmentally friendly:

- Places
- Food
- Business
- Public policy
- Societies

Agreed priority areas
Five overarching research areas were identified as priorities on the basis of their i) capacity to impact positively one or more aspects of the intersect between climate change and NCDs, ii)

amenability to mitigation through legal and ethical frameworks and governance systems and, iii) potential to contribute to addressing critical deficits in our knowledge and understanding of how to mitigate the current NCD and climate change crisis. They are:

1. ***Global and national policy***
   Can NCDs and climate change be integrated at the global and national level and how are NCD interventions justified by (and to) governments to assist them in balancing health and environmental gains versus restrictions on personal/corporate freedoms? This would require engaging all levels of government, civil service and civil society in systematically 'visioning' the goals of society globally and in determining which overarching principles have supremacy over others. For example, should health and/or climate change take precedence over economic growth or vice versa.

2. ***Food systems***
   How can food systems from 'paddock to plate / farm to fork' be re-engineered to maximise nutrition and minimise environmental harm. This would require researching:
   - the magnitude /extent of food waste
   - the utility of using food waste to make biofuels
   - the intergenerational safety and/or harms of genetically modified food
   - the impact on human health of food manufactured or produced with nanotechnology
   - the benefits and harms for NCDs, the environment and local economies of localising food production and consumption
   - the relative effectiveness of various strategies for averting the anticipated global food crisis eg containing population growth versus reducing overconsumption.

3. ***Corporate social responsibility*** (CSR)
   How can corporations be incentivised and enabled to deliver an integrated approach to improving health (specifically with regard to measures of NCD risk) and reducing carbon emissions and environmental damage more broadly?
   - do CSR principles and rhetoric match what corporations do in reality?
   - can/should governments and agencies such as the International Standards Organisation set stricter obligations for corporations to integrate and report on environmental and health impacts/outcomes as a condition of their registration?
   - should international trade law agreements include more specific exceptions allowing and protecting public and environmental interest regulation?

4. ***Health in all policies***
   How effective is a 'health in all policies approach' in mitigating NCDs and climate change? How do various government sectors understand, address, operationalise considerations of climate change and NCDs within their mandate? What influences them to do so? This would include the development of reliable audit tools to monitor each sector's response to health and climate change.

5. ***Regulatory interventions***
   What is the impact on behaviour change of specific regulatory interventions eg:
   - would mandatory labelling of foods with their carbon footprint change people's purchasing behaviour in favour of health and environmental considerations?
   - does mixed density housing reduce vectors of climate change and NCDs?

Additional critical issues were identified around urbanisation, social inequity /distributive justice, adaptation to climate change and dwindling natural resources. These, together with the areas above, are being refined into specific research questions through an international Delphi Process designed to canvass the expertise, insights and professional wisdom of researchers from the broad spectrum of disciplines working at the interface between NCDs and climate change.

Delegates at the 2nd WUN Global Health Justice Network Workshop were drawn from the following institutions and organisations: Australian National University, National Heart Foundation Australia, NSW Health Department, Australia, Union for International Cancer Control, Penn State University USA, The University of Sydney Australia, University of Alberta Canada, University of Bristol UK, University of Leeds UK, University of Queensland Australia, University of Western Australia, University of the West Indies. For enquiries please contact:

Associate Professor Ruth Colagiuri: ruth.colagiuri@sydney.edu.au
University of Sydney Medical Foundation Fellow

Figure S3 – Delphi Questionnaire Round 1

DELPHI SURVEY ON
RESEARCH PRIORITIES FOR NON-COMMUNICABLE DISEASE PREVENTION AND CLIMATE CHANGE

ROUND 1 – OCTOBER 11, 2011

Dear Colleague

Thank you for participating in this Delphi process which aims to expand on and complete the list of research priorities around the interface between climate change and non-communicable disease (NCD) prevention which was identified at the Worldwide Universities Network (WUN) Global Health Justice Network workshop held in Sydney, Australia, in May of this year. These five research priorities are shown in the *WUN Communiqué on Combining Climate Change and NCD Prevention* (see Appendix 1: page 2 of this survey document). We are seeking your views on additional areas of importance which may have been overlooked at the May workshop – particularly those which may be amenable to modification through the application of laws and regulatory processes.

To undertake the survey please read and consider the 5 research priority areas identified in the May 2011 WUN Communiqué (Appendix1) and:

1. Indicate **what, if any, broad research priority areas you believe are missing from the WUN Communiqué?** (eg urban design, architecture and planning, fiscal and economic policy)

   ....

   ....

2. **For each new area you add** (for Point 1 above), **please suggest at least one but no more than three priority research questions** that are a) feasible to conduct and b) address one or more knowledge/evidence deficit inthe overlap between climate change and NCD prevention.

   ....

   ....

   ....

   ....

   ....

Please return your completed survey form to Sinead Boylan: sboylan@usyd.edu.au by Friday 28th October. Please contact Sinead or me ruth.colagiuri@sydney.edu.au if you require clarification or have queries about any aspect of the survey.

Thank you in advance for your participation

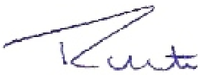

Associate Professor Ruth Colagiuri
October 11, 2011

Appendix 1

Worldwide Universities Network

International Diabetes Federation

THE UNIVERSITY OF SYDNEY

Communiqué on
Combining Climate Change and NCD Prevention
from the 2nd WUN Global Health Justice Network Workshop
held 5-7 May 2011 - Sydney, Australia

Rationale

Non-communicable diseases (NCDs) are not only a health problem, they are a sustainability and human development disaster. They are inextricably linked to the global environmental and economic crisis. Their impact on human capital is inexorable.

The United Nations General Assembly's unanimous decision in 2010 to hold a UN High Level Meeting on NCDs in September 2011 signals growing government recognition and concern about the impact of the global emergency arising from diabetes, heart disease and stroke, cancers and chronic respiratory diseases and their modifiable antecedent risks. These diseases are closely associated with mental illness and result in untold personal suffering and grave social and economic hardship. Individually and collectively they are major causes of poverty and lost productivity and pose a significant barrier, not only to human development, but also to global economic stability and prosperity.

These diseases form a substantial component of a broader global sustainability predicament that threatens economic and environmental sustainability and human capital. Their modifiable causes, particularly physical inactivity and under-or over-nutrition, go hand in hand with social inequality and with vectors of climate change such as over-urbanisation, poor urban design, sedentary automated work and play, and unhealthy food grown or manufactured through carbon intense food production methods. Many aspects of these things can be mitigated or ameliorated through laws. Yet, despite the obvious direct and indirect links between NCDs and climate change, the confluence remains under-examined in both global health and climate change research. Consequently, the Global Health Justice Network of the Worldwide Universities Network (WUN) met in Sydney on 5-7 May 2011 to consider how these issues might be addressed.

Purpose

The meeting brought together academic researchers from a range of disciplines including law, medicine and public health, the social sciences, environmental science, nutrition, psychology, policy, politics, and philosophy from academic institutions in the UK, the USA, Canada, the West Indies, and Australia. Its purpose was to explore, debate and identify key research deficits and priorities at the interface between NCDs and climate change to which the law might be applied to mitigate current and/or future harms and achieve co-benefits.

Process

Drawing on the 2008 *Sydney Resolution* (www.oxha.org) which called for urgent action on NCDs and their environmental vectors, delegates considered knowledge and evidence deficits that currently hinder progress on NCDs and climate change and exacerbate social injustice. The debate was anchored in the elements and components of optimally healthy and environmentally friendly:

- Places
- Food
- Business
- Public policy
- Societies

Agreed priority areas

Five overarching research areas were identified as priorities on the basis of their i) capacity to impact positively one or more aspects of the intersect between climate change and NCDs, ii) amenability to mitigation through legal and ethical frameworks and governance systems and, iii) potential to contribute to addressing critical deficits in our knowledge and understanding of how to mitigate the current NCD and climate change crisis. They are:

4. ***Global and national policy***
   Can NCDs and climate change be integrated at the global and national level and how are NCD interventions justified by (and to) governments to assist them in balancing health and environmental gains versus restrictions on personal/corporate freedoms? This would require engaging all levels of government, civil service and civil society in systematically 'visioning' the goals of society globally and in determining which overarching principles have supremacy over others. For example, should health and/or climate change take precedence over economic growth or vice versa.

5. ***Food systems***
   How can food systems from 'paddock to plate / farm to fork' be re-engineered to maximise nutrition and minimise environmental harm. This would require researching:
   - the magnitude /extent of food waste
   - the utility of using food waste to make biofuels
   - the intergenerational safety and/or harms of genetically modified food
   - the impact on human health of food manufactured or produced with nanotechnology
   - the benefits and harms for NCDs, the environment and local economies of localising food production and consumption
   - the relative effectiveness of various strategies for averting the anticipated global food crisis eg containing population growth versus reducing overconsumption.

6. ***Corporate social responsibility*** (CSR)
   How can corporations be incentivised and enabled to deliver an integrated approach to improving health (specifically with regard to measures of NCD risk) and reducing carbon emissions and environmental damage more broadly?
   - do CSR principles and rhetoric match what corporations do in reality?
   - can/should governments and agencies such as the International Standards Organisation set stricter obligations for corporations to integrate and report on environmental and health impacts/outcomes as a condition of their registration?
   - should international trade law agreements include more specific exceptions allowing and protecting public and environmental interest regulation?

7. ***Health in all policies***
   How effective is a 'health in all policies approach' in mitigating NCDs and climate change? How do various government sectors understand, address, operationalise considerations of climate change and NCDs within their mandate? What influences them to do so? This would include the development of reliable audit tools to monitor each sector's response to health and climate change.

8. ***Regulatory interventions***
   What is the impact on behaviour change of specific regulatory interventions eg:
   - would mandatory labelling of foods with their carbon footprint change people's purchasing behaviour in favour of health and environmental considerations?
   - does mixed density housing reduce vectors of climate change and NCDs?

Additional critical issues were identified around urbanisation, social inequity /distributive justice, adaptation to climate change and dwindling natural resources. These, together with the areas above, are being refined into specific research questions through an international Delphi Process designed to canvass the expertise, insights and professional wisdom of researchers from the broad spectrum of disciplines working at the interface between NCDs and climate change.

Delegates at the 2nd WUN Global Health Justice Network Workshop were drawn from the following institutions and organisations: Australian National University, National Heart Foundation Australia, NSW Health Department, Australia, Union for International Cancer Control, Penn State University USA, The University of Sydney Australia, University of Alberta Canada, University of Bristol UK, University of Leeds UK, University of Queensland Australia, University of Western Australia, University of the West Indies.

Figure S4 – Delphi Questionnaire Round 2

WORLDWIDE UNIVERSITIES NETWORK (WUN) GLOBAL HEALTH JUSTICE NETWORK
DELPHI SURVEY ON RESEARCH PRIORITIES FOR NON-COMMUNICABLE DISEASE PREVENTION AND CLIMATE CHANGE

Dear Colleagues

Many thanks for your generosity in responding to Round 1 (circulated to you in October/November 2011) of the above which identified 100 research questions under the following headings:

- Food systems & security
- Urban design, housing & transport
- Economics trade & business
- Social justice
- Behaviour, communication & information systems
- Regulation, governance, policy & systems

The results of Round 1 are presented below and we now seek your assistance in reducing and ranking these research questions and ask that you complete the survey form (below) and return it **by February 3, 2012 to Dr Sinead Boylan at sinead.boylan@sydney.edu.au**

In deciding which questions you wish to exclude and how the remaining questions should be ranked we ask that you keep in mind that we are primarily looking for research questions that:

- are of central importance to the global response to NCD prevention and climate change
- address a knowledge or evidence deficit that is preventing progress in area of high importance to the overlap between mitigating or adapting to climate change and preventing or significantly reducing the impact of NCDs and/or which focus in social and health justice issues
- address problems at the interface between NCDs and climate change which may be amenable to modification through legal interventions
- have some urgency or immediacy

Thank you in advance for your valuable input. Please do not hesitate to contact me ruth.colagiuri@sydney.edu.au or Sinead if you have any queries about the questions, the form, or the process.

Wishing you all a safe, happy, healthy and successful 2012,

Ruth Colagiuri

Associate Professor Ruth Colagiuri

WUN Research Priorities for Non-Communicable Disease Prevention & Climate Change: Delphi Survey Round 2`

Instructions: **Please tick (√) the options that most appropriately reflect your opinion noting that you do not need to rank priorities for any questions you exclude.**

Research question	Exclude	Include	Priority High	Med	Low
**Food systems and food security**					
**1.** What is the difference in nutritional properties between grass-fed and intensively reared livestock?					
**2.** How can food needs be defined to meet optimal nutrition within an environmentally sustainable agricultural program?					
**3.** What climate resilient fruits and vegetables crops could be promoted in different types of environments?					
**4.** How does climate change affect gene expression and production of functional food products in crops?					
**5.** How can we effectively predict crop production changes due to climate change and affects on food security (crop adaptation in regions most likely affected)?					
**6.** How does monoculture farming affect ecosystems health and the diet of local population in developing countries?					
**7.** Compared to confined animal feeding operations, do other agricultural practices have less impact on climate change? How are they brought about to a high production-level scale?					
**8.** What are the benefits of GM food?					
**9.** Which strategies are the most appropriate to improve food security through small-scale farming and environmentally sustainable food production?					
**10.** What impact does climate change have on the possibility that people have to eat fruits and vegetables?					
**11.** Should people eat fish – yes or no?					
**12.** General evidence that food security is not attainable without decreasing demand for unhealthy crop-intensive environmentally-damaging food.					
**13.** Access to potable water for human consumption and agriculture: what current best practices are needed to provide equitable access to water between geographic regions and usage demands? Are new best practices needed given climate change?					

WUN Research Priorities for Non-Communicable Disease Prevention & Climate Change: Delphi Survey Round 2`

Instructions: Please tick (√) the options that most appropriately reflect your opinion noting that you do not need to rank priorities for any questions you exclude.

Research question	Exclude	Include	Priority High	Med	Low
**14.** How do national agricultural policies affect urban food security in different regions?					
**15.** Should there be national guidelines to enable a diet good for both human and environmental health?					
**16.** What metrics, policies and governance structures are needed to address tough trade-offs needed in shifting agricultural patterns underway, especially aimed at reducing long term demand for meat and palm and developing a more diverse diet?					
**17.** What are effective incentives and disincentives for healthy and unhealthy food consumption which also promote low-carbon solutions?					
**18.** How does urban food security relate to the effectiveness of local transportation networks and market functioning in a country?					
**Urban design, transport and housing**					
**19.** What are the health impacts of a low carbon environment?					
**20.** How can housing policies contribute to climate change adaptation and mitigation?					
**21.** How can car-dependent cities shrink their carbon footprints in a way that reduces health inequalities?					
**22.** What are the health effects of innovative approaches to transport pricing?					
**23.** Does re-designing the built environment and transport systems to promote activity minimise the effects of climate change while promoting health?					
**24.** What role does walking or cycling to work have in reducing key NCD and what kinds of reductions in GHG would be expected?					
**25.** Quantify health co-benefits of alternative transportation plans for expanding cities, taking account both of air quality-related and physical activity related health benefits.					
**26.** What is the role of place in the determination of NCD prevention – is it additive, or independent of various other influences? (national and global contexts, policy food etc.)					
**27.** How can the health and climate change agendas be better aligned with those of urban planners and real estate developers?					

WUN Research Priorities for Non-Communicable Disease Prevention & Climate Change: Delphi Survey Round 2`

Instructions: Please tick (√) the options that most appropriately reflect your opinion noting that you do not need to rank priorities for any questions you exclude.

Research question	Exclude	Include	Priority High	Med	Low
**28.** Urban environment – what are the 'sweet spots' where economic success, climate mitigation and health promotion overlap?					
**29.** How does sustainable urban development and transport impact public health? What are the externalities that impact public health that have not been measured as of today? How do we measure these externalities?					
**30.** What are the methodologies to measure the impact of sustainable urban development and transport on health?					
**31.** How do patterns of urban land ownership and related land law impact on sustainable/healthy land use/transport policies and performance?					
**32.** As people are being encouraged to walk more in their communities, are they being exposed to CO2 and other pollutants from automobiles? Is there an ethical imperative here?					
**33.** Natural experimental analyses of how changes in physical living patterns and facilities have been reflected in behavioural and biomedical indicators of NCD risk.					
**34.** How can we integrate commitments to climate change prevention and mitigation into ongoing chronic disease prevention activities such as bikable/walkable communities, complete streets, and urban infill developments?					
**35.** Development and evaluation of the role and impacts of standards based on air quality guidelines, including standards/testing (for improved cook stoves, etc), and associated regulation.					
**36.** What are the most appropriate policies to shift from private motorized transport to healthier and cleaner transport alternatives?					
**37.** What comparative benefits for GHG emissions and prevention of NCD can a shift in motor sports to physical sports? What kind of regulation could be put forward to encourage such a shift?					
**38.** What are the political dynamics of shifting responsibility for land use planning from local to regional or state governments given the impending climate change crises? How successful could this be in a) changing practice on the ground when it comes to implementation and enforcement, and b) incorporating public health goals into regional plans?					
**39.** What are the relative effects of different policies on health in different urban locations with a range of disease burdens and transport infrastructure?					

WUN Research Priorities for Non-Communicable Disease Prevention & Climate Change: Delphi Survey Round 2`

Instructions: Please tick (√) the options that most appropriately reflect your opinion noting that you do not need to rank priorities for any questions you exclude.

Research question	Exclude	Include	Priority High	Med	Low
**40.** What is the relative importance of the health argument for influencing urban planning?					
**Economics, trade and business**					
**41.** What are the cost-benefits of fiscal policies such as a fat tax/ healthy subsidies, taking into account the potential benefits or costs that they might have in terms of environmental effects?					
**42.** How can econometric models that provide information about food prices be amended to incorporate information from environmental sensors that are used to warn of local food production declines?					
**43.** Valuation of the economic benefits of reduced NCD burdens (welfare and costs to health sector), of policy options that also reduce greenhouse gas emissions).					
**44.** What is the share of GDP in low middle income countries from car sales and FDI on food?					
**45.** What are the economic options for low middle income countries to promote healthy options?					
**46.** What are the economic options for major car industries to reduce their sales in low middle income countries?					
**47.** What is the economic impact related with health reported as: a) gains when a good policy/program/project is implemented; b) losses when no policy/program/project is implemented or when those implemented cause detrimental results?					
**48.** How does climate change and an increasing population affect urban economic growth and the affordability of food?					
**49.** Is it possible to describe or characterise the environmental costs (in terms of carbon emissions) of EU or US agricultural subsidies? This might need to be done at the level of individual commodities (e.g. oils, milk, fats etc)					
**50.** Is it possible to quantify the environmental costs (in terms of carbon emissions) or specific kinds of production subsidies?					
**51.** Is it possible to produce a combined analysis that analyses production subsidies in terms of both its impact on diet and on disease outcomes, and in terms of its impact on carbon emissions?					

WUN Research Priorities for Non-Communicable Disease Prevention & Climate Change: Delphi Survey Round 2`

Instructions: **Please tick (√) the options that most appropriately reflect your opinion noting that you do not need to rank priorities for any questions you exclude.**

Research question	Exclude	Include	Priority High	Med	Low
**52.** How connected are developing country capital city and regional markets to the international commodity markets?					
**53.** How can countries best evaluate the common impacts of climate change and NCDs of national and international trade?					
**54.** What joint mechanisms can countries put in place to reduce the negative impacts of trade on climate change and NCDs?					
**55.** What are some of the specific areas where the drivers of industry conduct are such that voluntary, CSR-led responses to the “problem” (which would need to be defined specifically) won’t go far enough? What are some of the areas where incentives and a greater alignment between the interests of industry and the specific policy goal (which would need to be defined specifically) mean that voluntary, CSR-led responses could work effectively?					
**56.** Can we develop a model of the areas where engagement with industry, and incentives, could yield effective CSR-led initiatives with a low regulatory cost? What are some of the areas where progress towards specific desired outcomes is likely to require regulation?					
**Social justice**					
**57.** Which are the best practices to combat indoor pollution in poor populations?					
**58.** Which are the technological, cultural and economic challenges for replacing bio-mass and coal stoves in poor populations of developing countries?					
**59.** Testing production, finance and delivery of technologies and solutions for the prevention of NCDs and climate change in issues that affect only the poor and the disenfranchised?					
**60.** The relationship between poverty, NCDs and climate change with macroeconomics and fiscal policy at the intersection between poverty and climate change.					
**61.** Research on the scenario as fiscal constraints increase as population ages and NCDs increase with limited capacity to address climate change. Decreased welfare transfers and increasing income inequality. What will be the regulatory mechanisms necessary to address such situations?					
**62.** Who bears the biggest burden of NCDs and climate change and why?					

WUN Research Priorities for Non-Communicable Disease Prevention & Climate Change: Delphi Survey Round 2`

Instructions: Please tick (√) the options that most appropriately reflect your opinion noting that you do not need to rank priorities for any questions you exclude.

Research question	Exclude	Include	Priority High	Priority Med	Priority Low
**63.** What mitigation measures need to be in place for the most vulnerable populations? What prevention measures are going to be the most effective?					
**64.** Linkage between climate change, poverty/social inclusion and NCDs.					
**65.** Do the small island countries in the Pacific represent some weight in the process or do they have to wait on big countries to change the rules?					
**66.** What is the capacity of people living in low socioeconomic conditions to address issues related to climate change and NCD prevention? Will they experience more of a burden?					
**67.** Will initiatives such as social marketing campaigns to educate the public about NCDs and climate change create knowledge gaps between low and high socioeconomic groups and thus contribute further to health inequities?					
**68.** How do women's gender roles related to climate change (e.g. use of solid fuel cooking stoves) increase their vulnerability to NCDS?					
**69.** Which policy and regulatory interventions to reduce indoor air pollution (use of solid fuels) can help achieve reduction in child mortality, promote gender equality/empower women and contribute to extreme poverty?					
**70.** How does climate change-driven famine/malnutrition increase the toll on the unborn/newborn to NCDs in adulthood?					
**71.**What statistical and process model frameworks can best capture the life chances and outcomes for individuals given early life events that are measurable?					
**72.** If all vegetables and fruits are produced locally, what could be the consequences on small island countries (economical, social and environmental)?					
**Behaviour, communication and information systems**					
**73.** What are the most effective ways to shift consumer demand from the 'western' diet to a more diverse predominantly plant-based diet?					
**74.** What incentives could be developed to encourage people to choose living locations that allow them to walk or cycle to work? What kinds of incentives could be used to encourage them to do so when they can?					

WUN Research Priorities for Non-Communicable Disease Prevention & Climate Change: Delphi Survey Round 2`

Instructions: Please tick (√) the options that most appropriately reflect your opinion noting that you do not need to rank priorities for any questions you exclude.

Research question	Exclude	Include	Priority High	Med	Low
**75.** What imperatives surround communication to people and publics, including responsibilities held by different parts of the media?					
**76.**How can the harms that lead to NCDs be described and conceptualised in a way that more easily supports policy formation?					
**77.** How do people rate the relative importance of climate change, household energy usage, consumer choices, and personal behaviours as influences on the risks of NCDs? How do they perceive the linkages, if any, between these multi-level factors? (also relate to factors relating to urban environments – design, congeniality, practicality, actual useability etc.					
**78.** Does evidence on the immediate health (NCD) benefits of more sustainable choices help to motivate individual behavioural change?					
**79.** Impact of greater awareness at all levels of society, including for the consumer (especially in the household context)					
**80.** How to change behaviour (as a collective rather than as individuals) to a highly active, low carbon transport system- (a question for primary research and for systems modelling). How to minimise rebound effects for a behaviour change?					
**81.** How can consumers get sufficient information to enable them to weigh up the evidence and advice?					
**82.** How can personal information on NCDs be most effectively collected, used and shared to inform national and global policy?					
**83.** How should individual and public interests in privacy be reconciled with the public interest in the use of personal information to develop policy and improve health?					
**84.** How can privacy law and administrative practice regulating the use of health information be harmonised globally?					
**85.** What are the information management systems and related technologies that can empower people to contribute to the information generation, dissemination and utilization e.g. how can mobile phones be used for that purpose?					
**86.** Can a suitable system be built through wrapped products that can be fed with data to be provided by users to enhance the services provided?					

WUN Research Priorities for Non-Communicable Disease Prevention & Climate Change: Delphi Survey Round 2`

Instructions: Please tick (√) the options that most appropriately reflect your opinion noting that you do not need to rank priorities for any questions you exclude.

Research question	Exclude	Include	Priority High	Med	Low
**Regulation, governance and policy**					
**87.** Both in the context of NCDs and mitigation of climate change, what are the priority areas where regulatory changes may be needed? How do those regulatory interventions propose to change the behaviours of populations, of business etc? What are the intermediate indicators that could be used to judge impact or success?					
**88.** What are the likely environmental effects of a fat tax/healthy subsidy to encourage healthier diet?					
**89.** How do the legal remits of government departments, local authorities, and health/education authorities affect the ability to pursue a healthy and sustainable land use/transport strategy?					
**90.** Public communication of questions concerning climate change and NCDs - what mechanisms of authority and accountability are engaged here, with what (if any) roles for democratic & other legitimising governance mechanisms?					
**91.** How should governments and other policy makers accommodate and respond to epistemic disputes, both in regard to sound science, and to the acceptability of political norms?					
**92.** What are some of the more promising models of inter-departmental structures that could be capable of delivering a genuine capacity to produce better alignment of policies for reducing NCDs and for tacking climate change (reducing overall greenhouse gas emissions) across sectors?					
**93.** What are the institutional features of successful examples of multi-sectoral approaches to policy development in other areas: ie in other areas where departments across all sectors were needed to improve the problem?					
**94.** Identifying effective models for implementation in the context of inter-sectoral action (health in all policies).					
**95.** How do we reduce the influence of irrationality and how do we agree on something internationally?					
**96.** What approaches (eg multi-criteria decision analysis) can support policy making at the interface of climate change and NCDs and to what extent?					
**97.**The use of main greenhouse gas emitting sectors (electricity generation, transport, housing, etc.) as an entry point for evaluation and policy intervention.					

WUN Research Priorities for Non-Communicable Disease Prevention & Climate Change: Delphi Survey Round 2`

<u>Instructions:</u> Please tick (√) the options that most appropriately reflect your opinion noting that you <u>do not</u> need to rank priorities for any questions you exclude.

Research question	Exclude	Include	Priority High	Med	Low
**98.** How to develop evaluation tools for social decision making that go beyond cost benefit analysis approaches?					
**99.** How do we shift policy and practices across disciplines and sectors?					
**100.** How can the range of interventions possible at multiple levels be understood as a whole of system intervention to optimise the potential for intervention at any level?					

Thank you for your time and input.

Please return your completed survey to Dr Sinead Boylan at

sinead.boylan@sydney.edu.au

Figure S5 – Table 4. Agreement (>90%) to exclude question as a research priority

Table 4. Agreement (>90%) to exclude question as a research priority.

**Question**	**Area**	**n**	**%**
How do we reduce the influence of irrationality and how do we agree on something internationally?	RGP	16	76%
Should people eat fish – yes or no?	F	15	71%
What comparative benefits for GHG emissions and prevention of NCD can a shift in motor sports to physical sports? What kind of regulation could be put forward to encourage such a shift?	U	14	67%
How can consumers get sufficient information to enable them to weigh up the evidence and advice?	BCI	14	67%
What are the economic options for major car industries to reduce their sales in low middle income countries?	ETB	13	62%
Do the small island countries in the Pacific represent some weight in the process or do they have to wait on big countries to change the rules?	SJ	13	62%
Will initiatives such as social marketing campaigns to educate the public about NCDs and climate change create knowledge gaps between low and high socioeconomic groups and thus contribute further to health inequities?	SJ	13	62%
How can privacy law and administrative practice regulating the use of health information be harmonised globally?	BCI	13	62%
Can a suitable system be built through wrapped products that can be fed with data to be provided by users to enhance the services provided?	BCI	13	62%

Key: BCI: Behaviour, communication and information systems; ETB: Economics, trade and business; F: Food; RGP: Regulation, governance, policy; SJ: Social justice; U: Urban design, transport and housing.

Figure S6 – Table 5. Agreement (>60%) to rank question as low priority

Table 5. Agreement (>60%) to rank question as low priority

Question	Area	n	%
What are the economic options for major car industries to reduce their sales in low middle income countries?	ETB	5	71%
What imperatives surround communication to people and publics, including responsibilities held by different parts of the media?	BCI	6	67%
Can a suitable system be built through wrapped products that can be fed with data to be provided by users to enhance the services provided?	BCI	4	67%
How can privacy law and administrative practice regulating the use of health information be harmonised globally?	BCI	5	63%
What are the information management systems and related technologies that can empower people to contribute to the information generation, dissemination and utilization e.g. how can mobile phones be used for that purpose?	BCI	8	62%

Key: BCI: Behaviour, communication and information systems; ETB: Economics, trade and business.
